# Re-constructing the river bed using the streamline-generation method

**DOI:** 10.1016/j.mex.2023.102539

**Published:** 2023-12-27

**Authors:** Zohre Aghamolaei, Masoud-Reza Hessami Kermani

**Affiliations:** Department of Civil Engineering, Faculty of Engineering, Shahid Bahonar University of Kerman, Kerman, Iran

**Keywords:** Finite difference method, Finite element method, Laplace equation, River bottom line, Streamlines, 2D hydraulic method, Finite Element Method and Finite Difference Method

## Abstract

River bed reconstruction plays an essential part in supporting the hydrodynamic simulation and understanding the morphological processes of a river. The streamlines can be solved using Laplace equations. The equation is first numerically solved in a computational environment and then adapted to the whole considered physical field to solve the resulting streamlines in a physical domain. One of the goals of this research is to determine the bottom line of the riverbed, and doing this through a field survey is very expensive, the methods presented in this research help a lot in reducing costs. By determining the concave line, the path of the flood in the river bed is determined, so one of the practical achievements and efficiencies of this research is flood trending at a low cost. In the present study, the bottom line is determined for the meandering Qinhe River, a distributary of the Yellow River, China, and Gaz River, located in Khuzestan Province, Iran. The method is based on the following steps:

• Reconstruction of 2D river based on the Laplace equation.
• Use the Finite Element Method to solve streamlines in the physical domain.
• Use the Finite Difference Method to solve streamlines in the physical domain.

## Highlights

• Reconstruction of 2D river based on the Laplace equation.
• Use finite element method to solve streamlines in physical domain.
• Use finite difference method to solve streamlines in physical domain.
• Predicted bottom line for the riverbed with hydrodynamic numerical models.

## Graphical abstract

**Figure:**
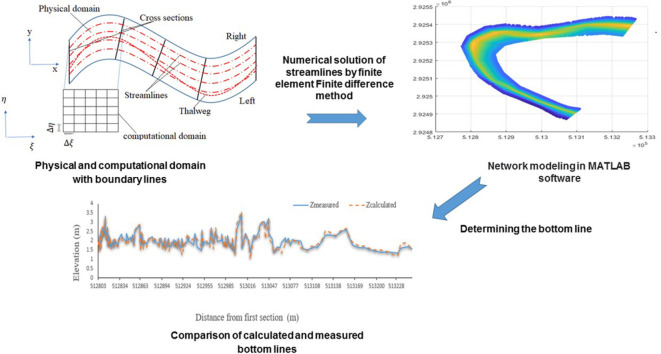

Specifications table

**Table:** 

Subject area:	Engineering
More specific subject area:	Hydrodynamic
Name of your method:	Finite Element Method and Finite Difference Method
Name and reference of original method:	L. Ruixun, W. Min, Zh. Xiaoli, H. Libing, Zh. Fangxiu, Y. Ming and W. Ming, Streamline-based method for reconstruction of complex braided river bathymetry, Journal of Hydrologic Engineering. (2021) 1084–0699.
Resource availability:	MATLAB Software

## Method details

River bed topography plays an important role in the numerical modeling of the flow, sediment transport, and morphology. One of the most important practical issues in this regard is free surface water flow under the effect of gravity, which includes tides, breaking waves on shallow beaches, and flood waves in rivers. One of the most fundamental assumptions in the derivations of the shallow water approximation theory is related to the pressure distribution in the hydrostatic state, and the results of this assumption are that the vertical acceleration of water particles has a negligible effect on the pressure. It can be said that the shallow water equations of the two-dimensional time-dependent system are nonlinear partial derivative equations of the hyperbolic type.

A numerical model is widely used to simulate the dynamics of a river as a function of flooded area, velocity, and depth. Numerical hydrodynamic models commonly used to simulate flooding are useful in hydraulic engineering and flood risk analysis [1]. It is necessary to justify model variables to support environmental challenges of modern engineering, such as water supply and flood risk assessment [2], [3], [4], [5], contaminant dispersion, disruption of freshwater habitats [6,7], sedimentation [8], and river morphology [9]. Accurate processing of the streambed in a numerical model highly affects the determination of the flow specifications. Several adequate advanced methods for finding river hydrography include multibeam acoustic tracking, bathymetric, aerospace, and remote sensing imaging methods. Multibeam acoustic tracking or so-called sonar systems determine river hydrography through active sensing, which provides surface topographic data and spatial resolution [10], [11], [12], [13], [14]. On the other hand, using a sonar system in shallow water environments is relatively rare due to difficulties in integrating data between wet and dry areas [15]. Thus, sonar measurement systems are primarily used in coastal and seabed applications [16]. Bathymetric and remote-sensing imaging systems detect visible and near-infrared radiation in the process of hydrography mapping in a river system. While it is possible to perform hydrometry remotely in reasonable sediment concentration levels, previous simulation studies indicated that these usages could be limited to shallow water and suffer from degrees of uncertainty [17], [18], [19], [20]. Although these approaches provide some hydrography data with high spatial and temporal accuracy, they are highly time-consuming and costly [21].

Le et al. (2020) stated that a set of measured elevation points and cross-sectional data might be the only means of depicting river topography in most cases [22]. These data serve as valuable records of river topography and can be employed to calibrate parameters such as roughness coefficient in practical and experimental cases. The development of interpolation algorithms for river bed reconstruction greatly depends on the availability of the data. These algorithms first generate an orthogonal curved grid confined to the body of water and the bottom line. Then, the measured cross-sectional data are applied to streamlines along the grid to interpolate the river hydrography [1,[23], [24], [25], [26], [27]]. The accuracy of hydrodynamic numerical models largely depends on the quality of the river bed model (especially along the main channel). Nevertheless, it is tough and costly to determine river hydrography in most situations; on the other hand, one suffers from a lack of available data to model the geometry of the river channel. The first step of river bed reconstruction is understanding river morphology, which focuses on the structure and shape of the river, including the river regime, channel geometry, and velocity profile distribution. River morphology is the result of the interaction of several factors, for instance, flow hydraulics (velocity, flow rate, roughness, and bed shear stress), channel characteristics (depth, width, shape, and slope), and upstream head (sedimentation and flow discharge). Horritt et al. (2016) studied two-dimensional shallow water flow in a channel considering the effects of grid quality and topographic data. They proved that grid quality affects modeling more than topographic data. They also stated that smaller grids can model the hydraulic features of rechargeable areas better and that a more accurate depiction of boundaries affects the accuracy of determining the features above [28]. Lai et al. (2013) presented a one-dimensional and a two-dimensional coupled hydrodynamic model for flow routing through a river watershed. The presented model has been used for large-scale hydrodynamic simulations during droughts and heavy rains. The results show that this model can make large-scale predictions and analyze flow considering important physical processes [29]. In [30], several linear spatial interpolation methods were presented based on which aerial maps of a field model of one computational domain can be categorized into two groups; firstly, topographic data are measured using common techniques like LiDAR or photogrammetry in flood plains where elevation models or contour lines are used to show the area's topography. Secondly, aerial river maps are of an area covered with water where river hydrography is difficult and expensive to determine via remote sensing techniques. Ruixon et al. (2021) presented a new approach based on produced streamlines using two-dimensional shallow water equations and interpolated measured cross-sectional data to reconstruct a river bed. They solved the produced grid using elliptic equations and determined the height of the inner points of a grid and the position of the bed river through interpolation [31]. Hilton et al. (2019) studied the reconstruction of the riverbed by measuring the height of the points and considering the coverage of dry and wet areas. This study suggested that, unlike existing techniques, this method can handle complex river morphologies such as islands and tributaries [32].

In 2019, Hilton et al. studied the reconstruction of the riverbed by measuring the height of the points and considering the coverage of dry and wet areas [32]. This study suggested that, unlike existing techniques, this method can handle complex river morphologies such as islands and tributaries. In most cases, measuring elevation points and cross-sectional data may be the only means of displaying river topography. Such data provide a good record of river topography and can be used to calibrate parameters such as roughness coefficient in practical applications.

Shokouhi (2012) used algorithms such as D8, D∞, RHO8, MFD, and DEMON to model the hydrodynamics of the river and flow routing. Based on the results of his work, it can be said that for all the studied algorithms, a threshold slope can be considered such that for slopes higher than 5 %, all five proposed algorithms are successful and determine the flow path well. Nevertheless, unrealistic currents are generated on lower slopes, which is problematic. Spatial interpolation methods are considered for many hydraulic fields. Many parameters affect the performance of these methods, but their effect is not fully understood [33]. Jin Li et al. (2011) conducted comparative spatial interpolation studies to evaluate the performance and reduce the effects of data properties on the performance of this method. They investigated inverse distance (IDW) and ordinary kriging (OK) methods for river hydrography. They further stated that increasing the range of data changes significantly impacts the method's performance, reducing its accuracy. On the other hand, after the necessary studies, it was found that if the distance between the data is variable, the method's accuracy will be improved. It was also found that the density of the data has little effect on improving the method's accuracy [34].

Schappi et al. (2010) stated that digital terrain models (DTMs) could be used directly to obtain the flood zone, but measuring river topography was difficult. Difficult aerial measurements only record the height of points relative to sea level; digital models cannot be used to obtain the area's topography. Therefore, they proposed a method for integrating topographic data in DTM. Although DTM, in most cases, assumes a regular distance between points, this solution is more beneficial for cross-sectional profiles on the left than for the longitudinal direction of the river. The algorithm presented in this study combines the river-side profile with DTM to generate a network and model the flow. The algorithm can also be used for linear river bed hydrography using linear interpolation, which is usually modified during the process by hydrological events such as the reconstruction of moving sections. This method shows how to reduce the error due to interpolation and how cross-sections limit the error rate. Finally, by defining break lines, they applied the proposed algorithm to the complex structure of the river [35].

Sayan Dey (2016) estimated channel hydrography using the River Channel Morphology Model (RCCM) based on the channel plan. In this study, the channel elevation model (DEM) is estimated based on the RCCM method and compared with the results of the LiDAR method [36]. Caviedes-Voullieme et al. (2014) studied the riverbed using one-dimensional interpolation of transverse sections. The present study that is trying to simulate floods mentioned that two-dimensional numerical simulations of river flows need large volumes of topographic data to construct an accurate digital terrain model that should cover the main river channel and potential flood area. DTMs for extensive floodplains are often produced by LiDAR; however, it is impossible to get LiDAR data from the riverbeds that are constantly flooded. The present study does not mention an information-generating algorithm in this section. This algorithm allows for the production of a riverbed that retains prominent morphological features like meanders and thalwegs. The results are analyzed from geometric and hydrodynamic perspectives with reasonable accuracy by performing two-dimensional simulations [37]. Baazm et al. (2022) used two-dimensional modeling of flooding using the finite element method (FEM). The achieved results from the finite element model are compared with the observational data at three stations [38].

Hessami Kermani et al. (2023), used the artificial intelligence method to investigate the bottom line for the meandering Qinhe River, a distributary of the Yellow River, China, and Gaz River, located in Khuzestan Province. The results of this research show the high accuracy of artificial intelligence methods in reconstructing the river bed and determining the depression line. [39].

Riverbed topography significantly influences on numerical models for flow, sediment transport, and morphology. This research focuses numerical modeling for riverbed reconstruction, aiming to determine riverbed hydrography and the Thalweg line. Comparison of methods provides insights into cost-effective flood trend prediction.

Previous studies proposed the finite difference method for solving hydrodynamic equations related to riverbeds. This article explores the accuracy of the finite element method, revealing its high precision and minimal error in addressing this matter.

## The process of riverbed reconstruction

**Fig. 1:**
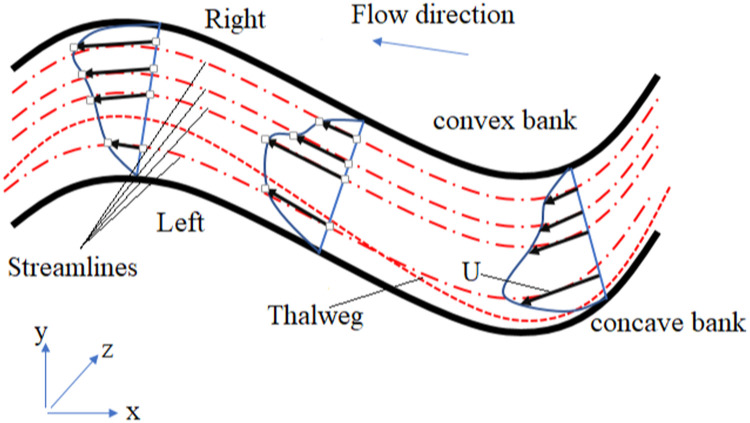
Flow lines and surface velocity.

To reconstruct the riverbed, the most critical step is to understand the morphology of the river. The morphology of a river focuses on the structure and shape of the river, channel geometry, and velocity profile distribution [40]. This process is the result of the interaction of several parameters, such as hydraulic flow (velocity, flow rate, roughness, and shear stress of the bed), channel characteristics (depth, width, shape, and slope), and upstream load (sediment and flow discharge) [41]. In natural rivers, the river pattern describes the plan, which includes straightness, meandering, or braiding [42]. Although straight and meandering rivers have similar morphology, the straight river has very little wave motion in the flood stage, and on the other hand, the meandering river is unstable against bending [43]. In braiding, the river surface is divided into several channels that may eventually merge. Fig. 1 shows a hypothetical river with a straight reach and a curved reach [42]. Maximum velocity streamlines at the flow level represent the average maximum vertical velocity along the river channel and divide the flow into two parts. The velocity streamlines pass at a maximum near the concave bend at each edge and approximately near the breakpoint between the bends [41]. The flow at the bottom of the river creates a high elevation at the concave bend and a low elevation at the convex bend. In bends, vertical velocity and transverse velocity affect the riverbed simultaneously. The vertical velocity distribution at the riverbed is similar to that at the surface, except that it is smaller. As shown in Fig. 1, the transverse velocity can transfer the bed load from the concave bend to the convex bend [40]. Therefore, the concave bend in the riverbed is constantly eroding while the transferred sediments are deposited in the convex bend. In addition, the cross-section of the riverbed in the concave bend can be considered a triangle with maximum water depth. At the bottom of the river, the thalweg is a line that shows the points with the lowest elevation along the river channel. As Fig. 1 shows, similar to the maximum velocity streamlines at the water surface, the thalweg path is close to the concave bend and moves from one bend to another downstream of the river. It can also be said that other curves on both sides of thalweg in the riverbed are used for hydrography of the main channel [40]. Two plates mark these curved lines on the bed. On horizontal planes, curved lines like streamlines are concentrated on the surface in a concave bend while scattered in a convex bend. In vertical planes, these curved lines are divided by measured cross-sections, and their vertices’ height is determined by interpolation. The height values of other vertices between cross-sections are obtained by linear interpolation. Therefore, one of the best methods for reconstructing riverbeds is the production of streamlines using the Thalweg route and cross-sections.

## Mathematical method of generating streamlines

**Fig. 2:**
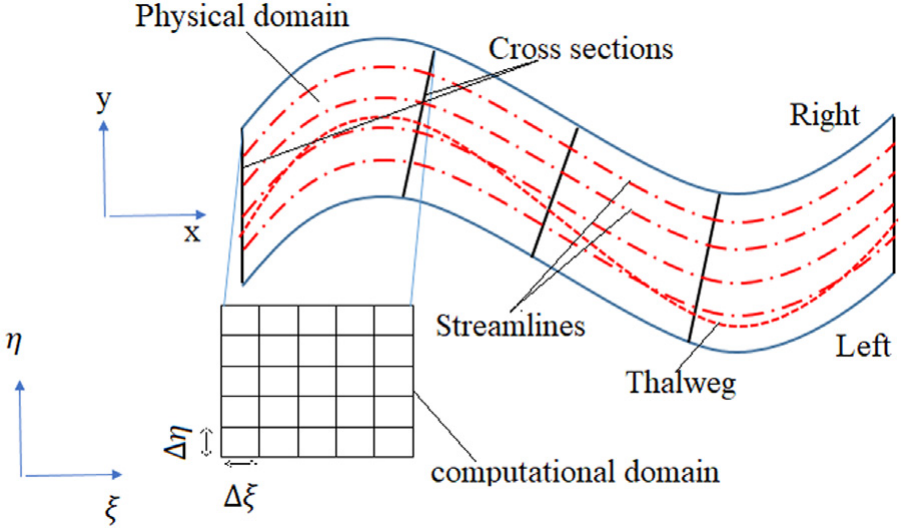
Physical and computational domain with boundary lines.

One solves the Laplace equation to produce streamlines based on the morphology of the river. Thompson et al. (1985) introduced streamlines based on elliptic equations and a numerical network [44]. Network generation is an advanced solution for obtaining an organized grid based on elliptic equations, widely used in computational hydraulics. In these algorithms, the Laplace equation can obtain the coordinates of points within the network and produce river boundary lines. The first step in producing a network is to delineate the river. One of the essential assumptions for using the Laplace equation to produce streamlines is that the water boundary, or the river boundary containing water, is considered the outer boundary, and the thalweg is the inner boundary. Also, to simplify the solution, we consider only grid lines along the main channel to obtain curved lines instead of considering grid lines across the river. Fig. 2 shows a physical and computational domain for generating a flow network to solve the Laplace equation. This computational network assumes that the thalweg and the water boundary are presented as boundary lines in the x and y coordinate system.

The purpose is to obtain the outlines of the streamlines at the river boundaries. The simple form of partial equations is the elliptical Laplace equation, which is defined in two-dimensional space as follows:

(1) $$\frac{\partial^{2}\xi }{\partial x^{2}}+\frac{\partial^{2}\xi }{\partial y^{2}}=0$$

(2) $$\frac{\partial^{2}\eta }{\partial x^{2}}+\frac{\partial^{2}\eta }{\partial y^{2}}=0$$

In these equations, x and y show the coordinates in the physical domain and ξ and η show the coordinates of the points in the computational domain.

Solving the Laplace equation using the finite element method and finite difference method is performed in a rectangular computational domain by considering a network with equal dimensions in two directions. As shown in Fig 2, the physical domain can be converted to a computational domain by dragging and rotating the boundaries. Thompson et al. (1977) mathematically justified the transformation of a physical domain into a computational domain by changing the independent and dependent variables, which by considering these transformations, Eqs. (1) and 2 become Eqs. (3) and 4 [45]:

(3) $$\alpha \frac{\partial^{2}x}{\partial \xi^{2}}-2\beta \frac{\partial^{2}x}{\partial \xi \partial \eta }+\gamma \frac{\partial^{2}x}{\partial \eta^{2}}=0$$

(4) $$\alpha \frac{\partial^{2}y}{\partial \xi^{2}}-2\beta \frac{\partial^{2}y}{\partial \xi \partial \eta }+\gamma \frac{\partial^{2}y}{\partial \eta^{2}}=0$$

(5) $$\alpha ={\left(\frac{\partial x}{\partial \eta }\right)}^{2}+{\left(\frac{\partial y}{\partial \eta }\right)}^{2}$$

(6) $$\beta =\frac{\partial x}{\partial \xi }\frac{\partial x}{\partial \eta }+\frac{\partial y}{\partial \xi }\frac{\partial y}{\partial \eta }$$

(7) $$\gamma ={\left(\frac{\partial x}{\partial \xi }\right)}^{2}+{\left(\frac{\partial y}{\partial \xi }\right)}^{2}$$

## Numerical solution of streamlines by finite element method

Based on the above, the differential equation governing streamlines can be considered as Eq. (1). This is defined using Galerkin's weighted residual theory according to Eq (8) [45]:

(8) $$\int \;\;\;\int \left(\frac{\partial^{2}\xi }{\partial x^{2}}+\frac{\partial^{2}\xi }{\partial y^{2}}\right)N_{i}dA=0\;i=1.2$$

(9) $$\int \;\;\;\int_{A}g\left(divf\right)dA=\int_{c}g.F^{T}.ndc-\int \;\;\;\int_{A}{\left(\nabla g\right)}^{T}.F.dA$$

Considering Green's theorem, we have:

(10) $$\;F^{T}=\left[\frac{\partial \xi }{\partial x}\;\frac{\partial \xi }{\partial y}\right]\;g=N_{i}\;\nabla g=\left[\frac{\partial N_{i}}{\partial x}\;\frac{\partial N_{i}}{\partial y}\right]$$

(11) $$\int \;\;\;\int_{A}\left(\frac{\partial^{2}\xi }{\partial x^{2}}\right)N_{i\;}dA=\int_{c}\frac{\partial \xi }{\partial x}N_{i}\;nx\;dc-\int \;\;\;\int_{A}\frac{\partial \xi }{\partial x}\;\frac{\partial N_{i}}{\partial x}dA$$

(12) $$\int \;\;\;\int \left(\frac{\partial^{2}\xi }{\partial y^{2}}\right)N_{i\;}dA=\int_{c}\frac{\partial \xi }{\partial y}N_{i\;}ny\;dc-\int \;\;\;\int_{A}\frac{\partial \xi }{\partial y}\;\frac{\partial N_{i}}{\partial y}d$$

Therefore, the weighted residual is equal to the following:

(13) $$\int_{c}\left(\frac{\partial \xi }{\partial x}nx+\frac{\partial \xi }{\partial y}ny\right)N_{i}dc+\int \;\;\;\int_{A}\left(-\frac{\partial \xi }{\partial x}\;\frac{\partial N_{i}}{\partial x}-\frac{\partial \xi }{\partial y}\;\frac{\partial N_{i}}{\partial y}\right)dA\;=0$$

The first integral represents the boundary conditions, which consist of two parts: a) around the specified natural boundary conditions (Cn), and b) around the specified essential boundary conditions.

(14) $$\int_{c_{e}}\left(\frac{\partial \varphi }{\partial x}nx+\frac{\partial \varphi }{\partial y}ny\right)N_{i}dc+\int_{c_{n}}\left(\frac{\partial \varphi }{\partial x}nx+\frac{\partial \varphi }{\partial y}ny\right)N_{i}dc+\int \;\;\;\int_{A}\left(-\frac{\partial \varphi }{\partial x}\;\frac{\partial N_{i}}{\partial x}-\frac{\partial \varphi }{\partial y}\;\frac{\partial N_{i}}{\partial y}\right)dA=0$$

The first integral, which represents the essential boundary conditions, is zero due to the residual weight method. Moreover, the second integral is zero according to the problem's natural boundary conditions. Considering the boundary conditions:

(15) $$\int \;\;\;\int \left(\frac{\partial \xi }{\partial x}\;\frac{\partial N_{i}}{\partial x}+\frac{\partial \xi }{\partial y}\;\frac{\partial N_{i}}{\partial y}\right)dA=0$$

(16)

(17) $$\frac{\partial \xi }{\partial x}=\left(\frac{\partial \xi_{1}}{\partial x}\;\frac{\partial \xi_{2}}{\partial x}\;\cdots \;\frac{\partial \xi_{n}}{\partial x}\right)\left[\begin{array}{c} \xi_{1}\\ \begin{array}{c} \xi_{2}\\ \begin{array}{c} \vdots \\ \xi_{n} \end{array} \end{array} \end{array}\right]=B_{x}^{T}.d$$

(18) $$\frac{\partial \xi }{\partial y}=\left(\frac{\partial \xi_{1}}{\partial y}\;\frac{\partial \xi_{2}}{\partial y}\;\cdots \;\frac{\partial \xi_{n}}{\partial y}\right)\left[\begin{array}{c} \xi_{1}\\ \begin{array}{c} \xi_{2}\\ \begin{array}{c} \vdots \\ \xi_{n} \end{array} \end{array} \end{array}\right]=B_{y}^{T}.d$$

(19) $$C=\left[\begin{array}{cc} K_{x} & 0\\ 0 & K_{y} \end{array}\right]$$

(20) $$\int \;\;\;\int \left(\frac{\partial \xi }{\partial x}\;\frac{\partial N_{i}}{\partial x}+\frac{\partial \xi }{\partial y}\;\frac{\partial N_{i}}{\partial y}\right)dA=\int \;\;\;\int \left(B_{x}B_{x}^{T}d+\;B_{y}B_{y}^{T}d\;\right)dA=0$$

(21) $$B_{x}B_{x}^{T}+\;B_{y}B_{y}^{T}=\left[\begin{array}{c} B_{x}\;B_{y} \end{array}\right]\left[\begin{array}{c} B_{x}^{T}\\ B_{y}^{T} \end{array}\right]=BcB^{T}$$

(22) $$B^{T}=\left[\begin{array}{cccc} \frac{\partial N_{1}}{\partial x} & \frac{\partial N_{2}}{\partial x} & \cdots & \frac{\partial N_{n}}{\partial x}\\ \frac{\partial N_{1}}{\partial y} & \frac{\partial N_{2}}{\partial y} & \cdots & \frac{\partial N_{n}}{\partial y} \end{array}\right]$$

On account of considering the grid as a rectangle in the present study, and considering one degree of freedom for each node, which is a total of four degrees of freedom, it is possible to write four lines from Khayyam's triangle.

**Fig. 3:**
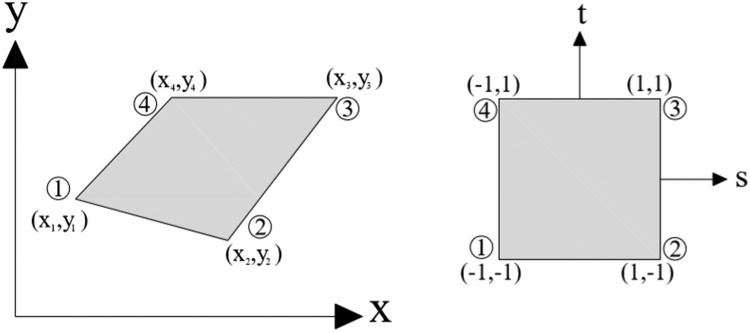
A four-node element intended to obtain relationships [45].

Polynomials according to Eq (23) and Eq (24) are also considered (Fig. 3):

(23) $$x=a_{1}+a_{2}s+a_{3}t+a_{4}st$$

(24) $$y=a_{5}+a_{6}s+a_{7}t+a_{8}st$$

(25) $$\left[\begin{array}{c} x_{1}\\ x_{2}\\ x_{3}\\ x_{4} \end{array}\right]=\left[\begin{array}{cccc} 1 & -1 & -1 & 1\\ 1 & 1 & -1 & -1\\ 1 & 1 & 1 & 1\\ 1 & -1 & +1 & -1 \end{array}\right]\left[\begin{array}{c} a_{1}\\ a_{2}\\ a_{3}\\ a_{4} \end{array}\right]$$

(26) $$\left[\begin{array}{c} \begin{array}{c} a_{1}\\ a_{2} \end{array}\\ \begin{array}{c} a_{3}\\ a_{4} \end{array} \end{array}\right]=\frac{1}{4}\left[\begin{array}{cccc} 1 & 1 & 1 & 1\\ -1 & 1 & 1 & -1\\ -1 & -1 & 1 & 1\\ 1 & -1 & 1 & -1 \end{array}\right]\left[\begin{array}{c} x_{1}\\ x_{2}\\ x_{3}\\ x_{4} \end{array}\right]$$

(27) $$x\left(s.t\right)=\left(1\;s\;t\;\text{st}\right)\frac{1}{4}\left[\begin{array}{cccc} 1 & 1 & 1 & 1\\ -1 & 1 & 1 & -1\\ -1 & -1 & 1 & 1\\ 1 & -1 & 1 & -1 \end{array}\right]\left[\begin{array}{c} x_{1}\\ x_{2}\\ x_{3}\\ x_{4} \end{array}\right]$$

(28) $$x=\left[\begin{array}{cccc} N_{1} & N_{2} & N_{3} & N_{4} \end{array}\;\;\;\right]\left[\begin{array}{c} x_{1}\\ x_{2}\\ \begin{array}{c} x_{3}\\ x_{4} \end{array} \end{array}\right]=N^{T}.d$$

(29) $$x\left(s.t\right)=N_{1}\left(s,t\right)x_{1}+N_{2}\left(s,t\right)x_{2}+N_{3}\left(s,t\right)x_{3}+N_{4}\left(s,t\right)x_{4}$$

(30) $$y\left(s.t\right)=N_{1}y_{1}+N_{2}y_{2}+N_{3}y_{3}+N_{4}y_{4}$$

(31) $$N_{1}\left(s.t\right)=\frac{1}{4}\left(1-s\right)\left(1-t\right)\;N_{3}\left(s,t\right)=\frac{1}{4}\left(1+s\right)\left(1+t\right)$$

(32) $$N_{3}\left(s.t\right)=\frac{1}{4}\left(1+s\right)\left(1-t\right)\;N_{4}\left(s,t\right)=\frac{1}{4}\left(1-s\right)\left(1+t\right)$$

(33) $$B=\left[\begin{array}{cccc} \frac{\partial N_{1}}{\partial x} & \frac{\partial N_{2}}{\partial x} & \frac{\partial N_{3}}{\partial x} & \frac{\partial N_{4}}{\partial x}\\ \frac{\partial N_{1}}{\partial y} & \frac{\partial N_{2}}{\partial y} & \frac{\partial N_{3}}{\partial y} & \frac{\partial N_{4}}{\partial y} \end{array}\right]$$

(34) $$\frac{\partial N_{i}}{\partial x}=\frac{\left|\begin{array}{cc} \frac{\partial N_{i}}{\partial s} & \frac{\partial y}{\partial s}\\ \frac{\partial N_{i}}{\partial t} & \frac{\partial y}{\partial t} \end{array}\right|}{\left|\begin{array}{cc} \frac{\partial x}{\partial s} & \frac{\partial y}{\partial s}\\ \frac{\partial x}{\partial t} & \frac{\partial y}{\partial t} \end{array}\right|}\;j=\left|\begin{array}{cc} \frac{\partial x}{\partial s} & \frac{\partial y}{\partial s}\\ \frac{\partial x}{\partial t} & \frac{\partial y}{\partial t} \end{array}\right|\;\frac{\partial N_{i}}{\partial y}=\frac{\left|\begin{array}{cc} \frac{\partial x}{\partial s} & \frac{\partial N_{i}}{\partial s}\\ \frac{\partial x}{\partial t} & \frac{\partial N_{i}}{\partial t} \end{array}\right|}{\left|\begin{array}{cc} \frac{\partial x}{\partial s} & \frac{\partial y}{\partial s}\\ \frac{\partial x}{\partial t} & \frac{\partial y}{\partial t} \end{array}\right|}$$

(35) $$a=\frac{\partial y}{\partial t}=\frac{1}{4}\left[y_{1}\left(s-1\right)+y_{2}\left(-s-1\right)+y_{3}\left(s+1\right)+y_{4}\left(1-s\right)\right]$$

(36) $$b=\frac{\partial y}{\partial s}=\frac{1}{4}\left[y_{1}\left(t-1\right)+y_{2}\left(1-t\right)+y_{3}\left(t+1\right)+y_{4}\left(-1-t\right)\right]$$

(37) $$c=\frac{\partial x}{\partial s}=\frac{1}{4}\left[x_{1}\left(t-1\right)+x_{2}\left(1-t\right)+x_{3}\left(t+1\right)+x_{4}\left(-1-t\right)\right]$$

(38) $$d=\frac{\partial x}{\partial t}=\frac{1}{4}\left[x_{1}\left(s-1\right)+x_{2}\left(-s-1\right)+x_{3}\left(s+1\right)+x_{4}\left(1-s\right)\right]$$

(39) $$\frac{\partial N_{1}}{\partial s}=\frac{1}{4}\left(t-1\right)\;\frac{\partial N_{1}}{\partial t}=\frac{1}{4}\left(s-1\right)$$

(40) $$\frac{\partial N_{2}}{\partial t}=\frac{1}{4}\left(1-t\right)\;\frac{\partial N_{2}}{\partial s}=\frac{1}{4}\left(-1-s\right)$$

(41) $$\frac{\partial N_{3}}{\partial s}=\frac{1}{4}\left(t+1\right)\;\frac{\partial N_{3}}{\partial t}=\frac{1}{4}\left(s+1\right)$$

(42) $$\frac{\partial N_{4}}{\partial s}=\frac{1}{4}\left(1-s\right)\;\frac{\partial \;N_{4}}{\partial t}=\frac{1}{4}\left(-1-t\right)$$

(43) $$B\left(s.t\right)=\frac{1}{\left|j\right|}\left[\begin{array}{ccc} B_{1} & B_{2} & \begin{array}{cc} B_{3} & B_{4} \end{array} \end{array}\right]\;B_{i}=\left[\begin{array}{c} a\frac{\partial N_{i}}{\partial s}-b\frac{\partial N_{i}}{\partial t}\\ c\frac{\partial N_{i}}{\partial t}-d\frac{\partial N_{i}}{\partial s} \end{array}\right]$$

(44) $$k=\int_{-1}^{1}\int_{-1}^{1}B^{T}CB\left|j\right|dsdt$$

If using the two-point Gaussian method to solve the above integral:

(45) $$k=\int_{-1}^{1}\int_{-1}^{1}B^{T}CB\left|j\right|dsdt=\sum_{i=1}^{4}\sum_{j=1}^{4}B^{T}CB\left|j\right|w_{i}w_{j}$$

(46) $$s_{i}=t_{i}=\pm \frac{1}{\sqrt{3}}\;w_{i}=w_{j}=1$$

After obtaining the values of ξ at the Gaussian points, these values can be transferred to the corner points of the element with the following matrix:

(47) $$T=\left[\begin{array}{cccc} 1+\frac{\sqrt{3}}{2} & -\frac{1}{2} & -\frac{1}{2} & 1-\frac{\sqrt{3}}{2}\\ -\frac{1}{2} & 1-\frac{\sqrt{3}}{2} & 1+\frac{\sqrt{3}}{2} & -\frac{1}{2}\\ 1-\frac{\sqrt{3}}{2} & -\frac{1}{2} & -\frac{1}{2} & 1+\frac{\sqrt{3}}{2}\\ -\frac{1}{2} & 1+\frac{\sqrt{3}}{2} & 1-\frac{\sqrt{3}}{2} & -\frac{1}{2} \end{array}\right]$$

## Numerical solution of flow lines with finite difference method

For numerical solution, Eqs. (3) and 4 are discretized by the finite difference method with a second-order central approximation:

(48) $$\alpha \left(\frac{x_{i+1.j}-2x_{i.j}+x_{i-1.j}}{{\left(\Delta \xi \right)}^{2}}\right)-2\beta \left(\frac{x_{i+1.j+1}-x_{i+1.j-1}+x_{i-1.j-1}-x_{i-1.j+1}}{4\Delta \xi \Delta \eta }\right)+\gamma \left(\frac{x_{i.j+1}-2x_{i.j}+x_{ij-1}}{{\left(\Delta \eta \right)}^{2}}\right)=0$$

(49) $$\alpha \left(\frac{y_{i+1.j}-2y_{i.j}+y_{i-1.j}}{{\left(\Delta \xi \right)}^{2}}\right)-2\beta \left(\frac{y_{i+1.j+1}-y_{i+1.j-1}+y_{i-1.j-1}-y_{i-1.j+1}}{4\Delta \xi \Delta \eta }\right)+\gamma \left(\frac{y_{i.j+1}-2y_{i.j}+y_{ij-1}}{{\left(\Delta \eta \right)}^{2}}\right)=0$$

Direct or iterative methods can be used to solve Eq (8) and Eq (9). The main differences between the two methods are time consumption and calculation amount. These two factors are less in the iterative solution method, and the calculation time has become shorter. The Gauss-Seidel algorithm is one of the most widely used iterative methods. This method calculates the values of adjacent points w, depending on computational values. As indicated before, one of the most prominent advantages of the present method is the fast convergence and reduced computational time. The Gauss-Seidel equations for Eq (48) and Eq (49) are as follows:

(50) $$2\left(\frac{\alpha }{{\left(\Delta \xi \right)}^{2}}+\frac{\gamma }{{\left(\Delta \eta \right)}^{2}}\right)x_{i.j}=\frac{\alpha }{{\left(\Delta \xi \right)}^{2}}\left(x_{i+1.j}+x_{i-1.j}\right)+\frac{\gamma }{{\left(\Delta \eta \right)}^{2}}\left(x_{i.j+1}+x_{i.j-1}\right)-\frac{\beta }{2\Delta \xi \Delta \eta }\left(x_{i+1.j+1}-x_{i+1.j-1}+x_{i-1.j-1}-x_{i-1.j+1}\right)$$

(51) $$2\left(\frac{\alpha }{{\left(\Delta \xi \right)}^{2}}+\frac{\gamma }{{\left(\Delta \eta \right)}^{2}}\right)y_{i.j}=\frac{\alpha }{{\left(\Delta \xi \right)}^{2}}\left(y_{i+1.j}+y_{i-1.j}\right)+\frac{\gamma }{{\left(\Delta \eta \right)}^{2}}\left(y_{i.j+1}+y_{i.j-1}\right)-\frac{\beta }{2\Delta \xi \Delta \eta }\left(y_{i+1.j+1}-y_{i+1.j-1}+y_{i-1.j-1}-y_{i-1.j+1}\right)$$

To start the calculation, the distribution of the x and y coordinates of the network points in the physical domain is obtained from an algebraic model in the computational domain. The x and y values are equal to the initial guessed values. In the next step, the variable values are replaced with the previous iterations and continue until a specific convergence criterion is reached.

### Interpolation of streamlines’ height

**Fig. 4:**
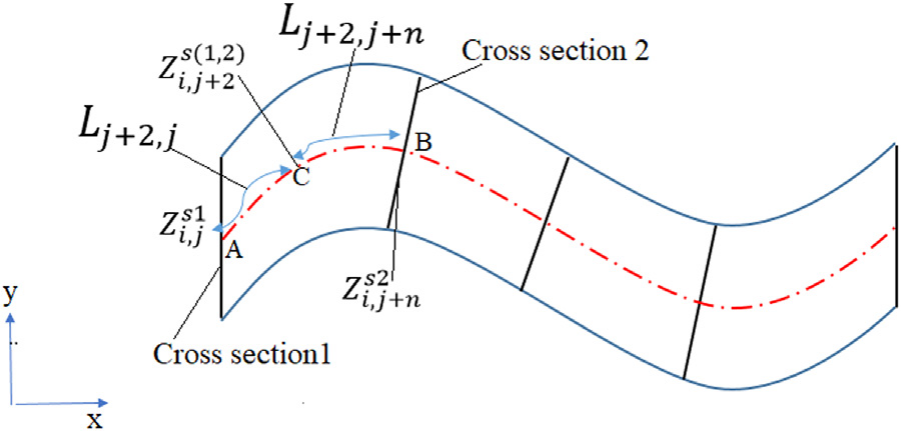
Interpolation of the height of riverbed points between two cross-sections.

The height of the vertices along the streamlines is interpolated from the nearest cross sections. As shown in Fig. 4, for cross sections 1 and 2, points A and B are the intersections between the streamlines and the two cross sections. Point C is the vertex along the streamline. The height of points A, C, and B are denoted by $\;Z_{i.j}^{s1},\;Z_{i.j+2}^{s(1.2)}$ and $Z_{i.j+n}^{s2}$.

*i* = ith streamline across the river

*j* = jth vertex along the streamline

The distance of the curve from points A to points C and B is expressed by Lj+2,j, and Lj+2,*j* + *n* respectively. The height of points A to B is interpolated using the measured cross sections. Therefore, the height of point C is calculated as follows:

(52) $$Z_{i.j+2}^{s\left(1.2\right)}=Z_{i.j}^{s1}+\frac{L_{J+2.J}}{L_{j+2.j}+L_{j+2.j+n}}\left(Z_{i.j}^{s1}-Z_{i.j+n}^{s2}\right)$$

Therefore, the interpolated streamlines are integrated with the topographic data of the flood plain, which can be deduced from elevation points and contour lines. The entire land-river model, including river channel and floodplain, is represented by a regular rectangular grid, usually a graphical information system (GIS) for the land model. Furthermore, multi-data forms such as dots, contour lines, or polygons can be used [40].

## Methods and materials

The finite element and finite difference methods are numerical methods for achieving approximate solutions to many physical and engineering problems.

The finite element method is a numerical method with a differential equation expressing governing behavior. In this method, smooth and continuous functions are used to approximate the desired unknown quantity. The main goal of the finite element method is to solve a complex problem by replacing it with a simpler model. The method considers the solution region as a set of small interconnected sub-regions called elements or finite elements. An approximate solution is assumed for each element in the following. By assembling these elements and the general equilibrium conditions of the system, the desired parameter for the computational range of the problem is calculated.

The function derivatives are approximated by their equivalent differences in the finite difference method to have the best solution. The finite difference method has been developed for solving partial differential equations based on calculating and storing information on network nodes. This is done by networking and applying the governing equations to each network node. We should mention that the nature of the equation is based on its properties. (For example, for an elliptic equation machine), the resulting algebraic equations must be solved simultaneously with the equation boundary values. However, the corresponding algebraic ones would be independently solved for parabolic equations. Different methods for numerical solutions of partial differential equations have been developed, classified into two categories: explicit and implicit. Through the discretization process, issues, including stability, consistency, and truncation errors in solving equations, have emerged that must be considered.

In the present study, the aim is to obtain the thalweg of Qinhe River, a tributary of the Yellow River in China, and Gaz River located in Khuzestan Province, Iran, in a computational range considering a rectangular element and seven cross sections using the finite element and finite difference method.

## Problem-solving algorithm

**Fig. 5:**
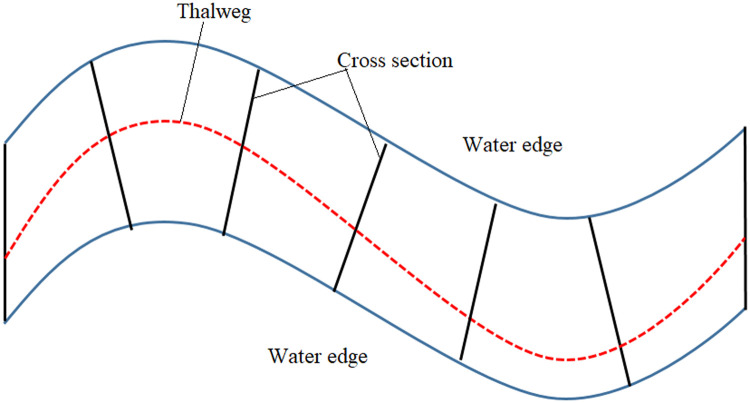
Display of cross sections for two case studies.

**Fig. 6:**
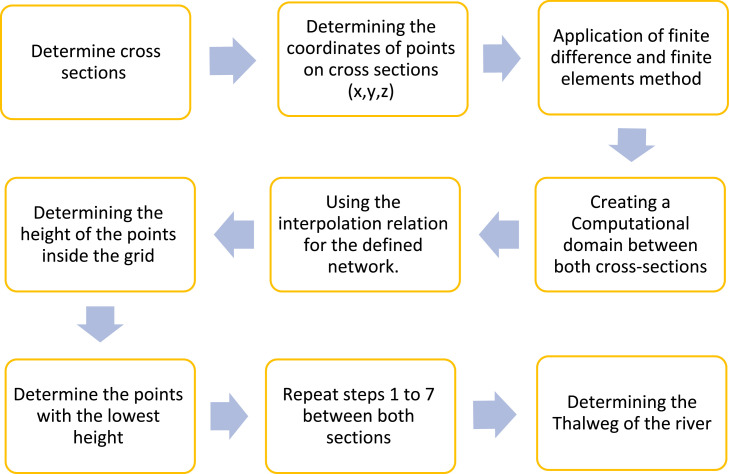
Problem-solving algorithm.

To determine the thalweg in the present research, firstly, several cross sections are considered along the river as in Fig. 5, and according to Fig. 2, the computational grid between every two sections is made through the two methods mentioned above. Then, using the data on the number of points of each cross section (X, Y, Z), the interpolation relationship (Eq. (52)), and Fig. 4, the height of the points between every two cross sections inside the grid is determined. Finally, by repeating the process for the entire studied area, the position corresponding to the lowest height of the points between cross sections indicates the location of thalweg (Fig. 6).

## Data selection

In this research, the coordinates of the points on every cross-section are used as the input data to determine the river's thalweg. Therefore, using the Global Mapper and AutoCAD Civil 3D software, the three-dimensional coordinates (x,y,z) of the points located on cross sections are read under the geographical location of the river. Subsequently, the depth and position of the line are determined based on the steps in sections 1–3.

## Studied areas

In this study, the presented method has been used to reconstruct two studied areas. To calculate the thalweg of the riverbed in each case study, a two-dimensional numerical model will be applied so that first, the topography of the river area is modeled using the finite element method, and the streamlines are calculated and then determined by interpolation of the thalweg of the river.

**Fig. 7:**
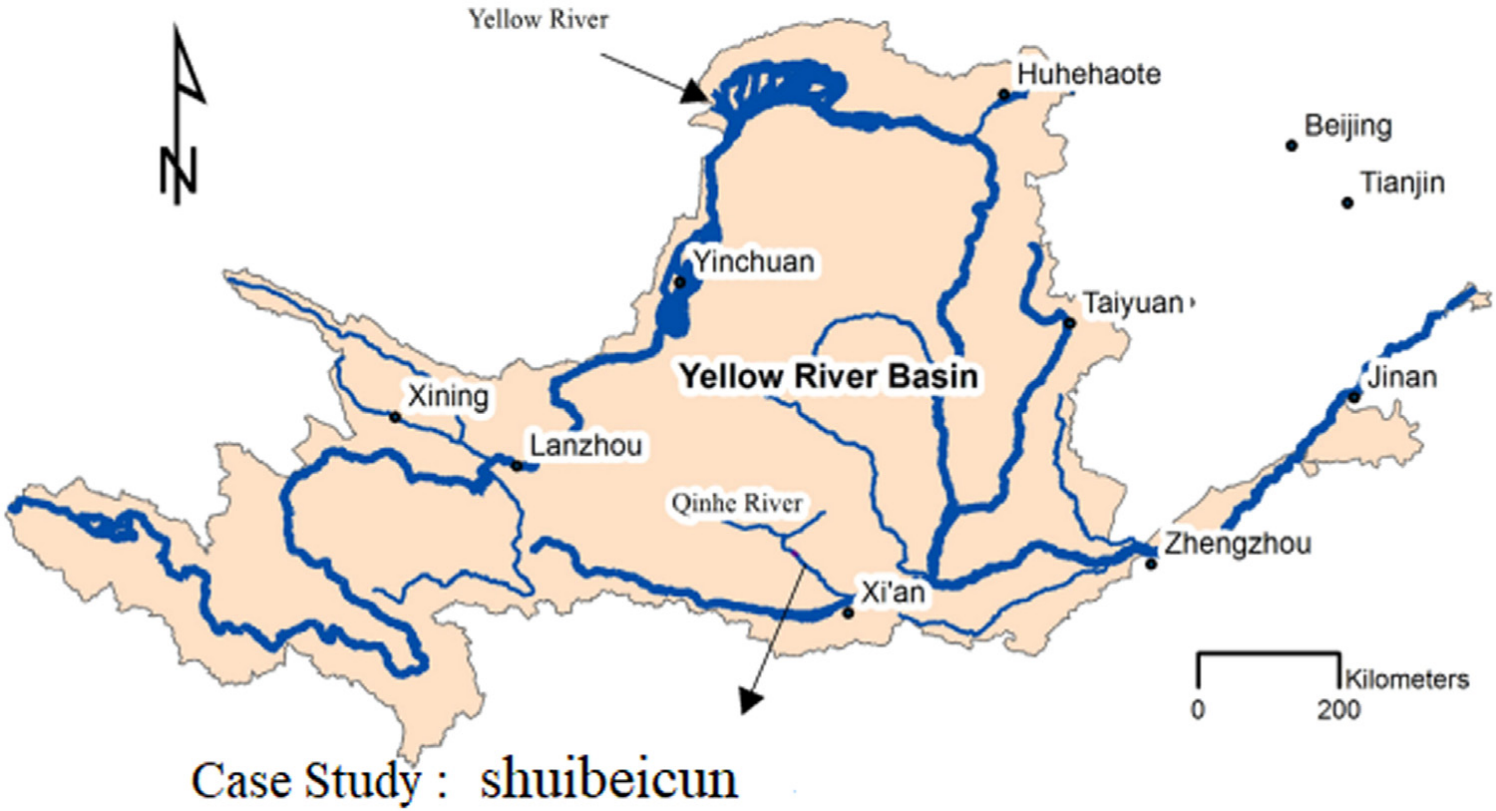
The first case study is the Qinhe River, a tributary of the Yellow River in China.

The first case study is the Qinhe River, a tributary of the Yellow River in China (Fig. 7).

**Fig. 8:**
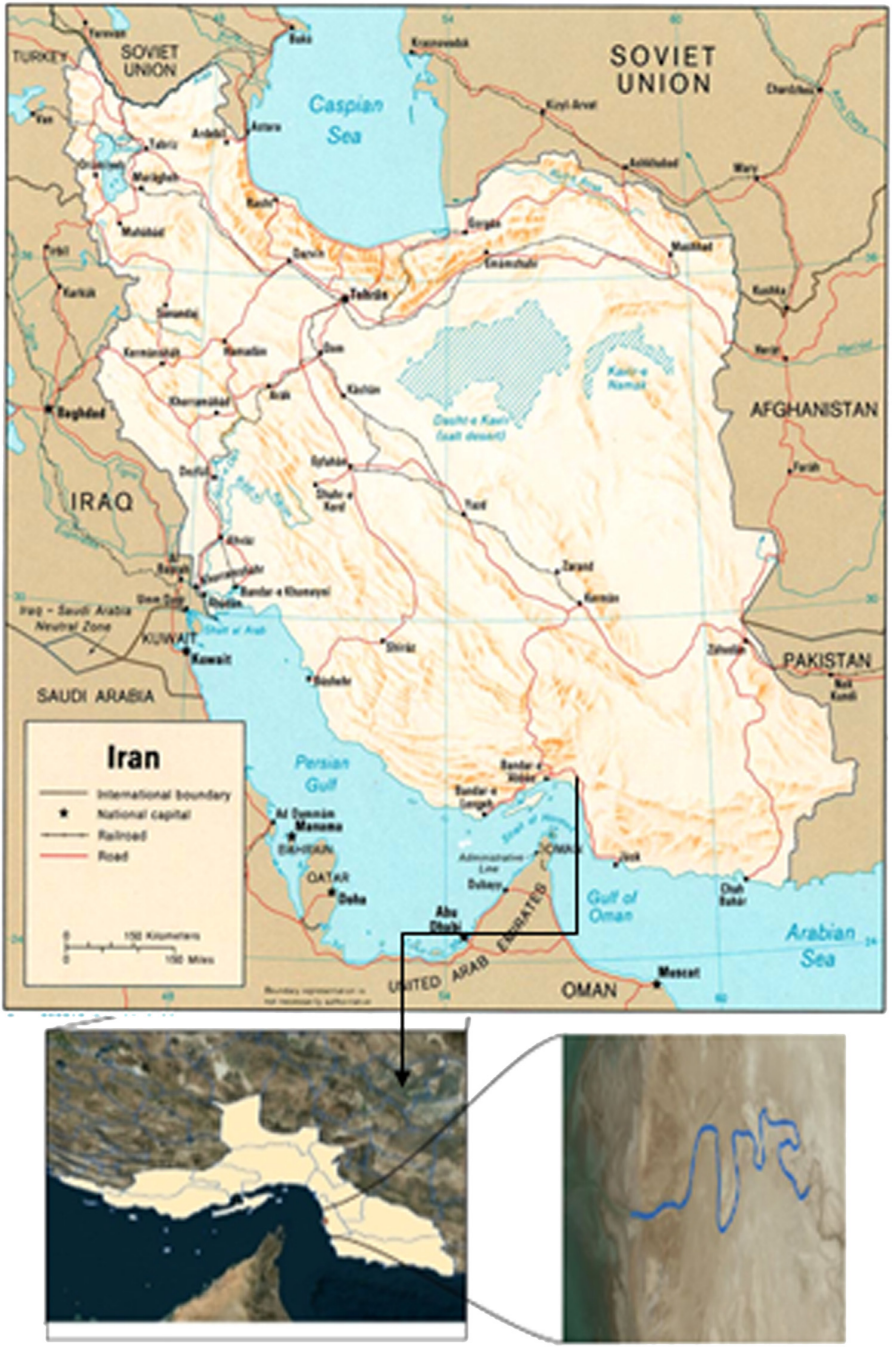
The second case study is a part of the Gaz River, located in Khuzestan Province, Iran.

The second case study is a part of the Gaz River, with a length of 22 km, located in Khuzestan Province, Iran. The Gaz River is 55 km long and is considered the main drainage of the Gaz drainage basin, which originates from the Bashagard Mountains and flows into the Oman Sea. The topographic information of the river used in this study was collected through the field survey (camera mapping) method. Fig. 8 shows the Gaz basin and river.

## Determining the boundaries of the water edge and thalweg

Fig. 5 shows the available data from the river in question, including the river's left and right banks, thalweg, and seven measured sections. Elevations and cross-sections were measured in September 2015. The thalweg is identified based on remote sensing images and measured sections. In September 2015, the river flow was about 30 m^3^ per second, and the river width was less than 50 m [40]. In addition, the lowest elevations of points from measured sections shown in Table 1 indicate the location of thalweg. Finally, thalweg can be identified by a function that passes through the lowest points and within river boundaries. The water edge was detected by solving one-dimensional open channel equations and measured streamlines. During the overflow period, the best condition for creating streamlines, an ideal water edge line is considered a boundary. Under these conditions, the channel is full of water without overflowing from the sides and has a maximum cross-sectional area [40]. Although the water edge should typically be obtained from field measurements, in most cases it is difficult to obtain this line in the event of flow overflow. One solution for calculating the water surface profile and identifying its intersection points in the geometric shape of measuring sections is to solve a one-dimensional open channel model. These intersection points are then used as key points to control the shape of the water's edge lines. Solving a one-dimensional open channel model requires two boundary the conditions. One of conditions is the overflow flow of the river, which is considered inlet flow and is estimated using the curvature of the cross-sectional area of section 1, which also corresponds to the outlet of the slope. The degree of the curvature depends on flow rate and water level and can be calculated statically using observed data. According to curvature of section 1, overflow discharge considering section geometry is equal to 6500 m^3^. The second boundary condition is the amount of roughness. Experimentally, roughness in the main channel is 0.032, and in the floodplain area is 0.035. Table 1 shows the obtained water level profile and locations of intersection points between seven sections and the water level profile, which decreases from 828.11 m in section 7 to 826.10 m in section 1. The intersection between the profile and cross section is measured from the left.

**Table 1:** Error calculation of Thalweg changes for two case studies.

Case studies	Qinhe river			Gaz river		
Method	Relative error	R	RMSE(m)	Relative error	R	RMSE(m)
Finite element method	25.3 %	0.879	1.63	27.2 %	0.987	1.72
Finite difference method	26.1%	0.871	1.929	27.4%	0.988	1.84

where n denotes the number of points along the river.

## Method validation

To calculate the thalweg of a riverbed, streamlines must first be determined. Left and right banks and seven measured sections are boundary conditions for product streamlines and computing networks. Maximum velocity streamlines pass near the concave bend at each edge and approximately near the breakpoint between bends. The flow at the bottom of the river creates high elevation at the concave bend and low elevation at the convex bend. In bends, vertical velocity and transverse velocity affect the riverbed simultaneously. The transverse velocity can transfer bed load from the concave bend to the convex bend. Therefore, the concave bend in the riverbed is constantly eroding while transferred sediments are deposited in the convex bend.

**Fig. 9:**
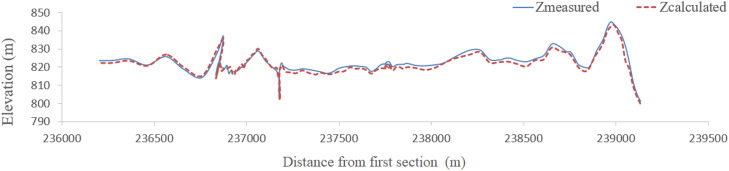
Comparison of calculated and measured bottom lines in the finite element method for the Qinhe River.

**Fig. 10:**
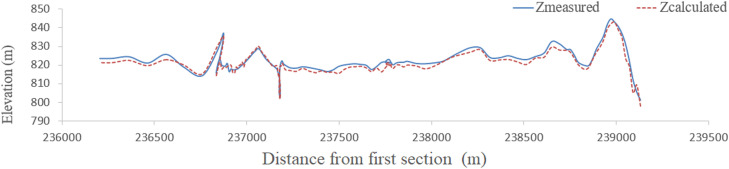
Comparison of calculated and measured bottom lines in the finite difference method for the Qinhe River.

**Fig. 11:**
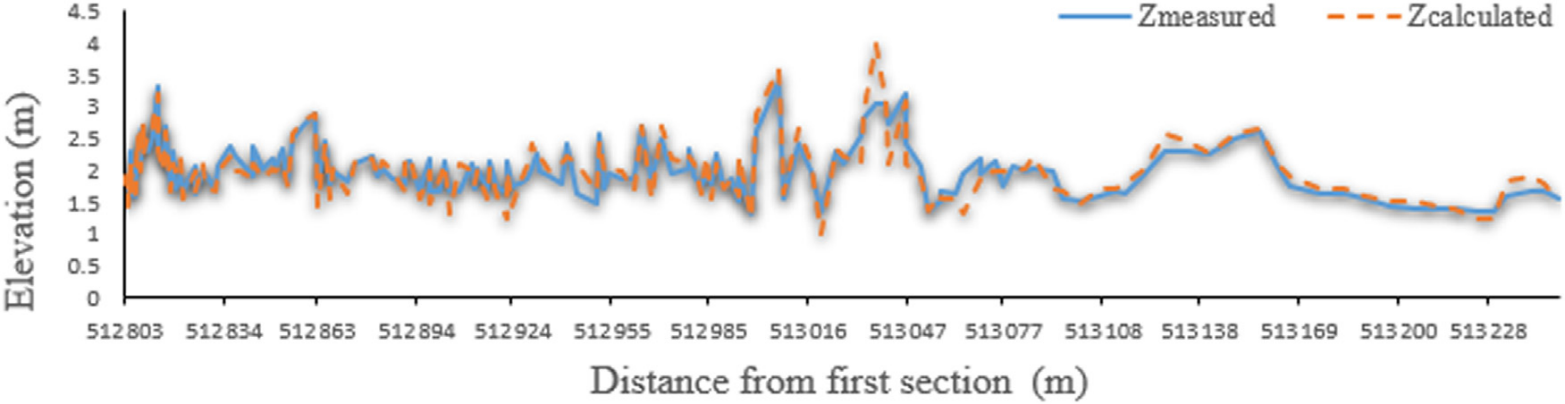
Comparison of calculated and measured bottom lines in the finite element method for the Gaz River.

**Fig. 12:**
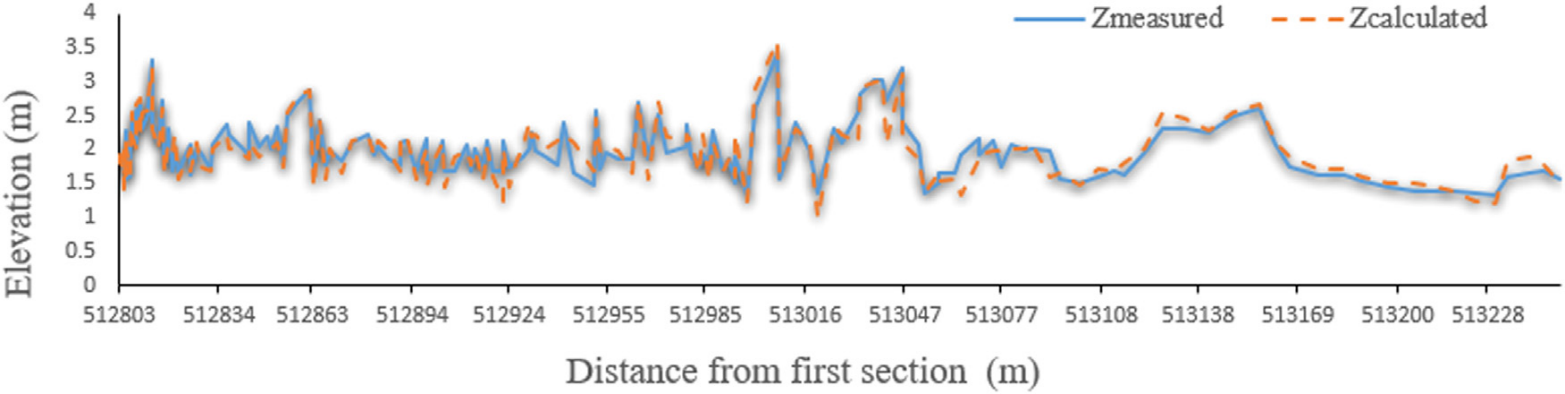
Comparison of calculated and measured bottom lines in the finite difference method for the Gaz River.

Finally, by generating streamlines, height values of other vertices between cross sections are obtained by linear interpolation. Since the thalweg of the river is the position of the points of bed that have the lowest height, the height of the points inside the calculation network of the thalweg of the river is determined by interpolation. Like maximum velocity streamlines at water level, the thalweg route is close to the concave bend and moves downward downriver. Therefore, one of the best methods for river bed reconstruction is the production of streamlines using high and low shores, and cross sections have been measured. Fig. 9, Fig. 10, Fig. 11, Fig. 12 compare thalweg calculated using finite element method with measured values. As can be seen, the present model has accurately predicted the thalweg of the riverbed.

## Calculate the model error

To calculate the error of the existing model, root mean square error (RMSE) and maximum relative error are used: Xmeasured represents the value obtained from the exact solution, while Xcalculated represents the value obtained from the existing model.

(53) $$\begin{array}{c} \text{RMSE}=\sqrt{\frac{\sum_{i=1}^{n}{\left(X_{\text{measured,}\;\text{i}}-X_{\text{calculated,}\;\text{i}}\right)}^{2}}{n}} \end{array}$$

(54) $$\begin{array}{c} \text{Relative}\;\text{error}\%=\sum_{i=1}^{n}\frac{\left|X_{\text{measured,}\;\text{i}}-X_{\text{calculated,}\;\text{i}}\right|}{X_{\text{measured,}\;\text{i}}}*100 \end{array}$$

## Discussion

In the presented research, the performance of models based on numerical methods has been compared using the data from two rivers in China and Iran. The results showed that the numerical methods in simulating the river bed and determining the bottom line are very accurate, on the other hand, the results of the finite element method in determining the bottom line were more accurate than the finite difference method. In general, it can be said that the use of numerical models in terms of cost, accuracy, and speed of predicting and determining the position of the riverbed has better performance than the measurement methods. Numerical methods are used to reconstruct the river bed when there is not much data available from the river bed, and in order to save time and money, instead of using direct methods, numerical methods are used for simulation. According to the announced results, it can be said that both methods presented in this research are highly accurate in their position to determine the characteristics of the river bed.

## Conclusion

An accurate geometric description of the main channel hydrography along the river is required to achieve acceptable results from numerical hydrodynamic models and understand essential river features such as discharge and riverbed shape. However, in many areas, we lack data to describe the geometry accurately, so numerical models can be used to estimate a river's thalweg. The present study presents a numerical model based on the finite element and finite difference method to determine the thalweg of the Qinhe River, a tributary of the Yellow River in China, and the Gaz River. The results show that the generated streamlines obtained by solving partial differential elliptic equations correspond well to the water-edge boundaries. One of the advantages of producing these streamlines is maintaining a constant shape of the interpolated cross sections and creating a smooth channel. The presented results show that if sufficient measurements of sections are available, the predicted thalweg for the riverbed is more consistent with the measured values by considering the upper and lower water edges as river boundaries. In the past research have been carried out to investigate the river bed using direct field measurement methods, finite difference methods, and artificial intelligence methods. In the current research, the finite element method has also been used to investigate the river bed and determine the bottom line, and by examining the results, it can be said that the accuracy of the modeling has been improved. Also determining the thalweg of the river bed by using direct field measurement methods is very expensive, the methods presented in this research help a lot in reducing costs. On the other hand, by determining the concave line, the path and extent of the flood in the river bed are determined, so one of the practical achievements and efficiencies of this research is flood trending at a low cost.

## CRediT authorship contribution statement

**Zohre Aghamolaei:** Conceptualization, Writing – original draft, Data curation, Formal analysis. **Masoud-Reza Hessami Kermani:** Writing – original draft, Writing – review & editing, Project administration, Supervision.

## Declaration of Competing Interest

The authors declare that they have no known competing financial interests or personal relationships that could have appeared to influence the work reported in this paper.

## Data Availability

Data will be made available on request.
